# Perspectives of Health Care Professionals on the Use of AI to Support Clinical Decision-Making in the Management of Multiple Long-Term Conditions: Interview Study

**DOI:** 10.2196/71980

**Published:** 2025-07-04

**Authors:** Jennifer Cooper, Shamil Haroon, Francesca Crowe, Krishnarajah Nirantharakumar, Thomas Jackson, Leah Fitzsimmons, Eleanor Hathaway, Sarah Flanagan, Tom Marshall, Louise J Jackson, Niluka Gunathilaka, Alexander D'Elia, Simon George Morris, Sheila Greenfield

**Affiliations:** 1 Department of Applied Health Science University of Birmingham Birmingham United Kingdom; 2 School of Life Course and Population Sciences King's College London London United Kingdom; 3 Department of Inflammation and Ageing University of Birmingham Birmingham United Kingdom; 4 Department of Metabolism and Systems Science University of Birmingham Birmingham United Kingdom

**Keywords:** artificial intelligence, AI, multiple long-term conditions, qualitative research, primary care

## Abstract

**Background:**

Managing multiple long-term conditions (MLTC) is complex. Clinical management guidelines are typically focused on individual conditions and lack a robust evidence base for patients with MLTC. MLTC management is largely delivered in primary care, where health care professionals (HCPs) have identified the need for more holistic yet efficient models of care that can address patients’ medical, pharmacological, social, and mental health needs. Artificial intelligence (AI) has proven effective in tackling complex, data-driven challenges in various fields, presenting significant opportunities for MLTC care. However, its role in managing patients with multifaceted psychosocial needs remains underexplored. The implementation of AI tools in this context introduces opportunities for innovation and challenges related to clinical appropriateness, trust, and ethical considerations. Understanding HCPs’ experiences of MLTC management and the factors influencing their attitudes toward using AI in complex clinical decision-making is crucial for successful implementation.

**Objective:**

We aimed to explore the perspectives of primary care HCPs on managing MLTC and their attitudes toward using AI tools to support clinical decision-making in MLTC care.

**Methods:**

In total, 20 HCPs, including general practitioners, geriatricians, nurses, and pharmacists, were interviewed. A patient case study was used to explore how an AI tool might alter the way in which participants approach clinical decision-making with a patient with MLTC. We derived concepts inductively from the interview transcripts and structured them according to the 5 categories of the model by Buck exploring determinants of attitudes toward AI. These included the *concerns* and *expectations* that contributed to the *minimum requirements* for HCPs to consider using an AI decision-making tool, as well as the *individual characteristics* and *environmental influences* determining their attitudes.

**Results:**

HCPs’ perspectives on managing MLTC were grouped into three main themes: (1) balancing multiple competing factors, including accounting for patients’ social circumstances; (2) managing polypharmacy; and (3) working beyond single-condition guidelines. HCPs typically expected that AI tools would improve the safety and quality of clinical decision-making. However, they expressed concerns about the impact on the therapeutic clinician-patient relationship that is fundamental to the care of patients with MLTC. The key prerequisites for clinicians adopting AI tools in this context included improving public and patient trust in AI, saving time and integrating with existing systems, and ensuring that the rationale behind a recommendation is apparent to enable a final decision made by an experienced human clinician.

**Conclusions:**

This is the first study to examine the attitudes of HCPs toward using AI decision-making tools in the context of managing MLTC. HCPs were optimistic about AI’s potential to improve decision-making safety and quality but emphasized that the human touch remains essential for patients with complex needs. We identified critical requirements for AI adoption, including addressing patients’ perceptions, time efficiency, and the preservation of clinician and patient autonomy.

**International Registered Report Identifier (IRRID):**

RR2-10.1136/bmjopen-2023-077156

## Introduction

### Background

Over 4 in 10 people >60 years are now living with multiple long-term conditions (MLTC) [1]. Effective management for patients with MLTC presents a complex set of challenges for health care professionals (HCPs). Clinical guidelines are typically focused on the management of individual conditions and based on evidence from clinical trials that, for pragmatic reasons, typically exclude patients with multiple comorbidities [2,3]. This often leads to patients accumulating heavy treatment burdens, involving complex daily medication regimens and coordination of multiple clinic appointments [4]. Most MLTC management takes place in primary care, and HCPs in this setting have identified the need for more holistic yet efficient models of care for these patients [5].

Artificial intelligence (AI) tools have been heralded as a potential solution to overcome the limitations in guidelines and evidence base for patients with MLTC [6]. Machine learning is a type of AI in which a computer with self-learning capacity can generate predictive algorithms and identify patterns from data in a way that mimics human intelligence [6,7]. This has been successful at tackling complex problems outside of health care [6]. The capacity to process large amounts of information systematically and reliably faster than the human brain makes the use of AI attractive for supporting the complex decision-making in the management of MLTC.

Despite significant interest in AI, it has so far had limited application in primary care settings [8]. There is growing interest and willingness among HCPs to use generative AI (eg, ChatGPT) in clinical practice; a recent survey of 1000 UK general practitioners (GPs) found that a fifth reported using it in clinical practice for tasks such as documenting clinical notes but also to consider differential diagnoses [9]. The Royal College of General Practitioners has recognized that there is vast potential for AI in general practice, particularly for administrative tasks but also in supporting decision-making [10]. However, policies advising AI software developers and HCPs on the safe and legal integration of AI tools into clinical practice are currently in their infancy, and insights from frontline HCPs should be harnessed. The impact of AI on diverse and marginalized populations, particularly concerning ethnicity and socioeconomic status, is a key area of concern in the field of AI [11]. Studies have shown that AI models can perpetuate existing biases; algorithms trained on nonrepresentative datasets may perform inadequately for underrepresented groups, potentially exacerbating health inequalities [11]. Addressing these challenges requires inclusive AI development practices and robust legal frameworks to ensure equitable health care delivery.

Previous studies exploring perspectives on AI have treated it as a broad and hypothetical idea [12]. However, technological development and media discourse have since moved on at pace. Exploration of current and informed perspectives using realistic examples of AI-based tools is needed. Insights from HCPs regarding the challenge of implementing AI tools are necessary for their effective integration into practice, especially in primary care, which involves multiple small organizations working independently and deals with a high burden of mental and chronic illness for which the use of clinical judgment may be more appropriate than a rigid clinical pathway [13].

### Objectives

This study forms part of the Optimising Therapies, Disease Trajectories, and Artificial Intelligence–Assisted Clinical Management for Patients Living With Complex Multimorbidity (OPTIMAL) research project, which aims to develop a predictive AI-based tool that can use existing health care data to identify and present the best treatment options for a patient with MLTC (hereafter referred to as the OPTIMAL tool). We aimed to explore the perspectives of HCPs on managing patients with MLTC in primary care and the value and feasibility of using AI tools to support decision-making with these patients.

## Methods

### Study Design

This study used qualitative methodology through an interpretive paradigm to explore the subjective experiences and meanings that HCPs assign to the idea of using AI tools in managing MLTC [14]. Schultze and Avital [15] have outlined methods to encourage the collection of rich data, including “grounding the interview in participants’ own experiences” and “providing an explicit framework for guiding the participants to articulate and interpret their experiences.” To apply these principles, we fostered a conversational atmosphere in 1:1 semistructured online interviews following a topic guide (Multimedia Appendix 1) that provided an explicit framework to allow the participants to describe and interpret their experiences freely. In designing the topic guide, we prompted participants to think about examples to illustrate their perspectives, with the aim of achieving more nuanced, in-depth responses.

**Figure 1:**
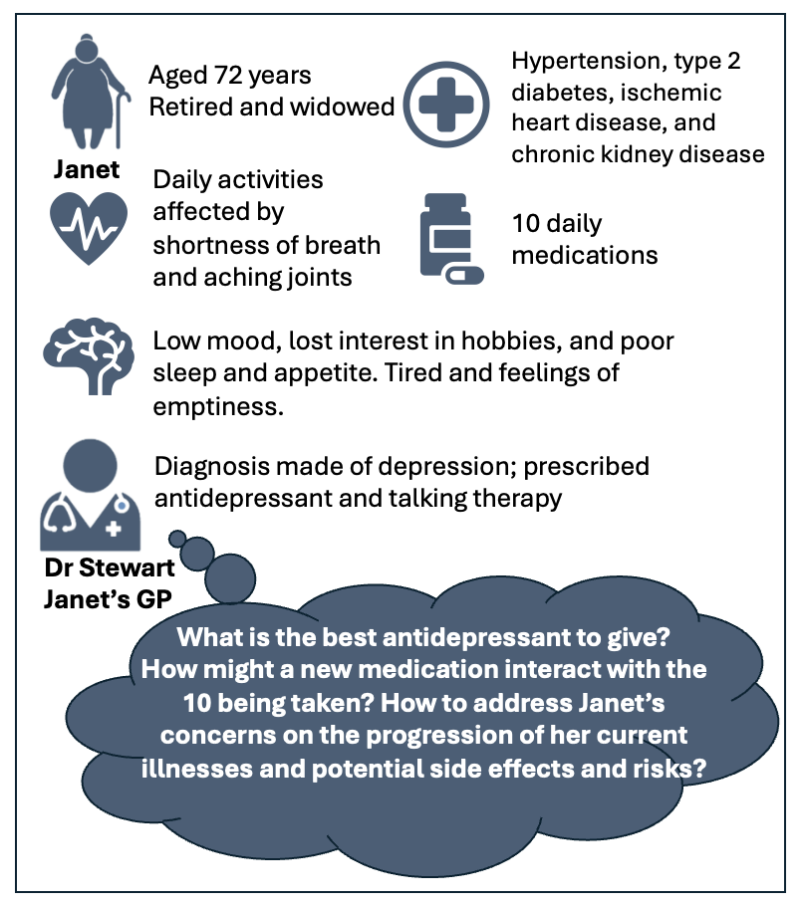
Patient case study and prescribing challenge in multiple long-term condition (MLTC) care. The following case study is typical of the challenges faced by health care professionals and patients in MLTC care: Janet’s general practitioner (GP) has diagnosed her with depression and suggested she undergo talking therapy and start an antidepressant medication. However, they are both concerned about potential interactions of antidepressants with her existing medications and the risk of progression of her existing medical conditions.

To ground participants’ responses in the context of their experiences of typical clinical practice, we co-designed a case study (Figure 1) with clinicians and patient advisory group members that reflected a common clinical decision (choosing an antidepressant) for Janet, a hypothetical patient with a common combination of both physical and mental MLTC [16]. Participants were asked to outline their usual decision-making process for prescribing an antidepressant for Janet and then consider using instead the outputs of the OPTIMAL tool to help inform their prescribing decision.

### Participants and Recruitment

We recruited HCPs working in the National Health Service (NHS) in the West Midlands region of the United Kingdom. Inclusion criteria for HCPs were current experience working in primary care or geriatrics with a role involving prescribing or medication reviews. Our primary focus was GPs. However, clinical pharmacists, geriatricians, and nurse prescribers were also included as they are involved in a significant proportion of MLTC management and their perspectives on AI in clinical practice have not been widely explored elsewhere [17]. We welcomed participants with and without previous experience with AI in health care. Recruitment was purposive based on recruiting a sample that reflected the aforementioned desired clinical roles. We also aimed for diversity in the age, years of experience, gender, and ethnicity of the participants. We aimed to recruit up to 30 participants or until data saturation was achieved, which was determined by the study team to be when no new information could be added to the framework of themes that emerged from the data [18,19].

HCPs were recruited via advertisements sent to national health care societies (including the British Geriatrics Society and Society for Academic Primary Care) and local social media and WhatsApp groups for HCPs. HCPs who were interested and made contact were then sent the study information sheet, background information on AI, case study, and consent forms (Multimedia Appendices 2 and 3).

### Interviews

A pilot interview was conducted with AD, a GP registrar who was not involved in the initial study design, to test content and processes. Responses from the pilot interview were not included in the analysis. Following the pilot interview and 2 participant interviews, the order of the topic guide was changed to bring the case study earlier as it became clear that participants typically did not have any previous experience using AI in health care and the example case helped frame the discussion in the context of MLTC. The case study was used verbatim for all GP participants but adapted post hoc for nurse practitioners and clinical pharmacists to a decision about diabetes or hypertension medication, which more closely reflected their typical clinical practice compared with prescribing an antidepressant.

Participants were able to choose their preferred form of interview (face-to-face, video, or telephone), and it took place in the private setting where they felt most comfortable, which was typically their own home or their clinical workplace. The interviews were audio recorded.

The interviewer and primary analyst (JC) was an academic GP with 10 years of experience in clinical practice. Participants were aware that they were being interviewed by a “fellow” GP and that the study was part of the interviewer’s PhD. Of the 20 participants, 2 (10%) were known by the interviewer before the interview through previous clinical training, whereas the remaining 18 (90%) were not known previously. A reflexive approach, documented through field notes, was used to consider how these factors may have influenced the findings.

### Patient and Public Involvement

People with lived experience of MLTC as patients or as carers have been involved throughout the OPTIMAL study. The 8 patient and public advisory group members have contributed to the research design (particularly regarding recruitment), development of the topic guide and participant-facing documents, and interpretation of the results.

### Ethical Considerations

Written consent was obtained via email before the interviews and reconfirmed in the interview. Participants were assured that their contribution was anonymous, and they were offered the opportunity to withdraw from the study for any reason for up to 2 weeks after the interview. Audio recordings were made using an encrypted digital recording device and stored securely at the University of Birmingham. Ethics approval was granted by the NHS Heath Research Authority (reference: 22/SC/0210). A £15 (US $20.38) shopping voucher was offered to participants in recognition of their contribution.

### Data Analysis

Audio recordings of the interviews were transcribed verbatim using a professional transcribing service. Deidentified transcripts were encrypted and analyzed thematically using the framework method in NVivo (version 12; QSR International) [20].

JC conducted all the interviews and coded all transcripts. To improve the validity of the coding framework, a second researcher, SF, independently coded a 10% sample of the transcripts. Rather than relying on statistical measures of interrater reliability, which can oversimplify the interpretive nature of qualitative data, the coding was reviewed using a discussion-based reconciliation process [21,22]. Differences in coding were examined collaboratively with the entire study research team, which helped ensure conceptual clarity, consistency, and analytic rigor while acknowledging the reflexive nature of qualitative analysis.

### Coding

Codes were assigned to the transcripts line by line, and the coding framework was derived inductively from the data. Similar codes were combined, and themes that emerged were applied to the analytical framework. After reviewing and revising among the study team in fortnightly meetings, the final themes were determined, and the interpretations were explored. A summary of overall themes from each interview was sent to participants for comment and was used for further discussion and refinement of the concepts and themes with the study research team and the patient advisory group.

**Figure 2:**
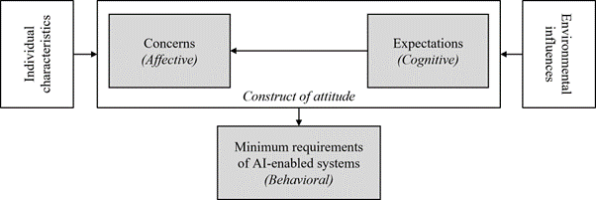
The model by Buck et al of the determinants of general practitioners’ attitudes toward artificial intelligence (AI)–enabled systems. This figure is reproduced under the terms of the Creative Commons Attribution license from work published in the Journal of Medical Internet Research.

First, codes and themes related to the current challenges in the management of MLTC were grouped together. Codes related to the use of AI were treated separately. The model by Buck et al [23] (Figure 2 [23]) was then used as a theoretical framework to explore and separate the affective, cognitive, and behavioral components of HCPs’ attitudes toward the use of AI in managing MLTC.

### Theoretical Framework

Theoretical frameworks allow for a deeper understanding of how people and the cultures and organizations they belong to operate, interact, and behave [24]. To explore the attitudes of GPs toward the use of AI in primary care in Germany, Buck et al [23] built their framework on the previous work by Rosenberg et al [25] on the construct of attitudes toward change.

The model by Buck et al [23] distinguished the affective (emotional reactions, empathy, and feelings), cognitive (ideas and knowledge), and behavioral (the extent to which attitudes predict actions and intentions to act) dimensions to GPs’ attitudes toward AI-enabled systems in health care. They also found that individual characteristics such as previous experience with AI and environmental influences such as the attitude of professional bodies also influenced GPs’ perspectives [23]. The model by Buck et al [23] is based only on GPs’ attitudes, and we interviewed a range of HCPs (also including nurses, pharmacists, and geriatricians). Given that the model by Buck et al [23] is based on the well-established work by Rosenberg [25] on attitudes toward change across multiple industries, we anticipated that the model by Buck et al [23] would apply equally well for the other groups of HCPs.

We assigned codes that we had inductively derived from the data to the framework by Buck et al [23] to determine how and in what ways the data fit the model and deepen the understanding of attitudes toward AI. Inductive and deductive analysis techniques complemented each other to achieve a more comprehensive understanding of barriers to the implementation of AI in managing MLTC in primary care [26]. Inductive reasoning was used to see what freely emerged, and the theory by Buck et al [23] was applied to explore this further through the lens of attitude toward change.

## Results

### Participants

A total of 33 HCPs responded to the advertisements, and 20 (61%) interviews took place (n=13, 39% of the HCPs expressed an initial interest but did not commit to an interview). Interview duration ranged from 23 to 63 (mean 37, SD 9) minutes. A total of 95% (19/20) of the participants opted for a video call, whereas 5% (1/20) preferred a telephone call. Following 20 interviews, we determined that we had reached data saturation, with sufficient diversity in participant characteristics. There was an even balance of men and women among the 13 GPs (n=6, 46% men; n=7, 54% women) and the 2 geriatricians (n=1, 50% of each gender). However, all the nurse and pharmacist participants were women (5/5, 100%). A total of 35% (7/20) of the participants reported being of White ethnicity, and 40% (8/20) reported being of Asian ethnicities. All participants were aged between 25 and 55 years, and most (13/20, 65%) had completed >10 years of clinical training. Most (18/20, 90%) had no or little previous experience of AI in health care, but 10% (2/20) of the participants, who were male GPs, were advisors to digital health companies developing AI for health care, and another male GP was involved in commissioning and regulating AI in health care. Table 1 further summarizes the participant characteristics.

**Table 1:** Characteristics of the participants (N=20).

Characteristic and subgroup			Participants, n (%)
**Gender**			
	Men	7 (35)	
	Women	13 (65)	
**Professional role**			
	GP^a^	11 (55)	
	GP registrar	2 (10)	
	Nurse practitioner	3 (15)	
	Prescribing pharmacist	2 (10)	
	Geriatrician	2 (10)	
**Ethnicity**			
	Asian	8 (40)	
	White	7 (35)	
	Did not answer	5 (25)	
**Age group (y)**			
	25-34	4 (20)	
	35-44	6 (30)	
	45-54	4 (20)	
	≥55	0 (0)	
	Did not answer	6 (30)	
**Time since completion of training (eg, finishing medical school; y)**			
	2-5	5 (25)	
	5-10	2 (10)	
	>10	13 (65)	

^a^GP: general practitioner.

### Overview

First, we inductively derived 3 main themes that summarized the challenges that HCPs currently face in managing MLTC: prioritizing multiple factors, managing polypharmacy, and practicing outside of usual guidelines. Throughout the Results section, we present the frequency of participants who discussed each theme or concept to provide full transparency regarding the level of consensus among the participants [27,28].

**Figure 3:**
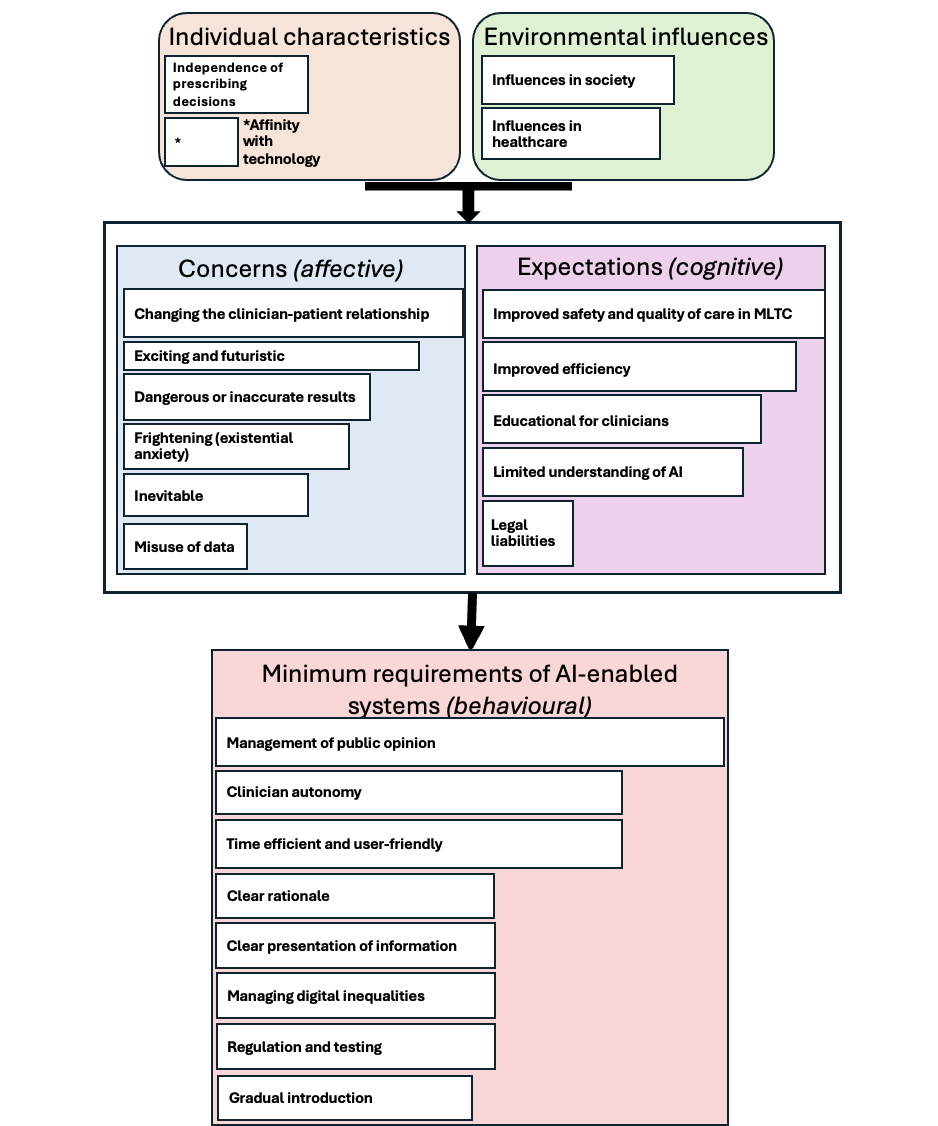
Summary of the 24 concepts that describe health care professionals’ attitudes toward using artificial intelligence (AI) to manage multiple long-term conditions (MLTC) applied to the 5 categories of the model by Buck et al. The width of the white boxes is proportional to the number of participants who discussed each theme.

Second, to describe HCPs’ attitudes toward using AI to manage MLTC, we discuss 24 concepts derived from the data as they applied to the 5 categories of the model by Buck et al [23]. HCPs explained their *concerns* (category 1), *expectations* (category 2), and the *minimum requirements* (category 3) for them to consider using an AI decision-making tool to manage MLTC. We also describe the *environmental influences* (category 4) and *individual characteristics* (category 5) that affect these attitudes. Figure 3 provides an overview of the 24 concepts as applied to the 5 categories, and we then describe these further supported by direct quotations from the data.

### Managing MLTC

There was strong consensus (18/20, 90% of the participants) that managing MLTC was an especially challenging aspect of clinical practice. The 3 most common themes for MLTC illustrated the complexity of that challenge: first, the difficulty of prioritizing multiple factors (20/20, 100%), including addressing both the patient and clinician priorities and managing physical and mental health conditions simultaneously; second, managing polypharmacy and addressing side effects and medication interactions (16/20, 80%); and third, that managing MLTC often necessitates practicing outside of single-condition guidelines (13/20, 65%). Each of these challenges was felt to be significantly compounded by the time constraints in general practice. Participants recognized the particular importance of providing holistic care for patients with MLTC accounting for their individual social circumstances, level of community or family support, health literacy, and any language barriers:

Multiple physical conditions isn’t too tricky, it’s when you get then mental health problems on top of physical health problems, and then particularly if you get disability such as dementia on top of that, and if you get social problems—isolation, loneliness, housing issues, domestic violence—then it becomes really complex.

P5; GP; man

There’s a lot of conflict between.... all of the NICE guidelines which are very individual condition specific. And in terms of clinical management, it’s trying to weigh up which is more important, depression or kidney failure. And you just can’t. There is no answer there. And trying to do that, if you’ve only got ten minutes to have that conversation...you can go to a problems list and there can just be thirty, forty items on there. Trying to weigh all that up, check through the medications list, again lots of medications yes difficult, challenging and also probably the most rewarding as well, because I think that’s where you as a GP, yes that is our specialism really, is trying to treat the whole patient and not just their individual conditions. It’s almost that kind of freedom and permission to not follow the guidelines or to just do what feels right and what seems right for the patient at the time.

P14; GP; man

### Attitudes Toward AI Decision Support in Patients With MLTC Using the Model

Figure 3 [23] summarizes the 24 concepts that describe HCPs’ attitudes toward using AI to manage MLTC as they applied to the 5 categories of the model by Buck et al [23].

#### Cognitive: Ideas, Knowledge, and Beliefs About AI

##### Improved Safety and Quality of Care in MLTC Management

All participants (20/20, 100%) anticipated that AI would be capable of dealing with multiple factors, including patient demographics, medical history, medications, and test results. Most participants felt that this would improve the quality and safety of decisions made with patients with MLTC (13/20, 65%) and that this could allow for best practice to be standardized:

There’s a tendency for decision support to be very one dimensional to one particular condition...it then becomes much, much, much more complex [with MLTC] and that’s where potential errors will come in...using AI to help guide decisions based on a bit of complexity...can help interpret for clinicians to help them make better decisions.

P3; GP; man

Many participants (14/20, 70%) discussed that they felt that AI could prevent clinicians from making mistakes by providing additional checks:

It might reduce the chance for human error...[with only 10 minutes] it’s easy to miss an interaction, a side effect, a key point in the patient’s history.

P2; GP; woman

In total, 30% (6/20) of the HCPs expressed that AI may allow for more holistic consultations and more tailored support, particularly in the context of MLTC:

[It] would be a personalised synthesis of available information and computions.

P14; GP; man

##### Improved Efficiency

A total of 90% (18/20) of the participants discussed tasks that they felt that AI may facilitate in general practice. In total, 40% (8/20) of the HCPs anticipated that AI would be generally time saving for clinicians and increase efficiency for the health system if it allowed more time for other tasks:

So, I guess the biggest advantage is probably that it would free up time eventually and free up human resources.

P4; GP; woman

Although our main focus was AI-based decision-making tools, participants also discussed a range of other clinical activities in which AI may play a role, including administrative tasks (eg, writing a referral letter or documenting a consultation; 6/20, 30%), summarizing information (7/20, 35%), diagnosis (3/20, 15%), triage (9/20, 45%), and interpreting radiology images or blood tests (6/20, 30%). Typically, clinicians felt that AI would be very competent at these tasks:

You get [AI] to write a lot of the supporting letters, save a lot of administration time, right?

P16; GP; man

When...you make a referral to hospital...especially with patients with multi-morbidity...I thought AI, if you could look through and just basically list these are their current problems, these are their current medications and it just wrote it out for you, kind of like chat GPT, that would be really timesaving I think for GPs.

P19; GP registrar

##### Educational for Clinicians

Most HCPs (16/20, 80%) expressed that an AI decision-making tool had the potential to be educational for clinicians. If the outputs were unexpected, they felt that this may prompt further learning:

For me it would be helpful to have some way...that you can just sort of understand what it is about the particular factors that has led the decision support tool to come up with that particular treatment...there’s a good opportunity there for learning and understanding.

P3; GP; man

##### Limited Understanding of AI

Participants typically felt unsure of exactly what AI is and how it differs from existing non-AI computer tools (15/20, 75%):

To me AI is almost something that is talked about and I don’t understand.

P8; nurse; woman

Participants typically had limited experience of the use of AI within the health care setting, and even those GPs who had experience working with AI technology companies did not feel that they fully understood AI. Participants did not dwell on the distinction between AI and existing non–AI-based computerized decision-making tools and templates. A total of 60% (12/20) of the participants referred to existing computerized tools such as QRISK for cardiovascular risk assessment or consultation templates. These participants felt that using AI was simply another method to support decision-making and may not feel like a significant change in practice:

Essentially, we’re just replacing something that already exists and instead of it being human intelligence on [a multidisciplinary team (MDT)], it’s artificial intelligence on an MDT isn’t it really?

P18; nurse practitioner; woman

In a sense we already use computers to do things like calculate scores for us, but that’s not a thinking-ulator [like AI] that’s just calculators.

P4; GP; woman

##### Legal Liability

In total, 15% (3/20) of the HCPs were concerned from a medicolegal perspective about the implications of not following a recommendation from an AI tool:

Who governs it and who takes responsibility for it, where’s the medicolegal side...I would only feel comfortable if they said the tech company takes liability if it gets it wrong! But if they...said “no, no, no, you’re liable,” then I would be like “look, you can amber/green/red flag it, right, but I don’t want you to make the decision”...you would want the clinician to be happy with the liabilities.

P16; GP; man

Contrastingly, 10% (2/20) of the HCPs described that AI tools may improve documentation of the rationale for decisions, which would be positive from a medicolegal perspective and could back up the clinician:

And then from a medicolegal perspective you can then have a clearer justification for why you’ve gone for a particular treatment...is definitely, definitely good I would say.

P3; GP; man

#### Affective: Emotional Reactions and Feelings About AI

Participants expressed mixed emotional reactions to the use of AI in managing MLTC.

##### Changing the HCP-Patient Relationship

The potential for an AI decision-making tool to alter the HCP-patient relationship was a universal concern for participants (20/20, 100%). A total of 85% (17/20) of the HCPs described how continuity of care and human empathy was especially important for patients with MLTC. One participant was extremely concerned that AI tools would replace interaction with patients. However, most HCPs (14/20, 70%) felt that it was unrealistic that an AI tool would replace them entirely due to the high therapeutic value the HCPs placed on human interaction:

...people want that human approach, human understanding...and AI will take this completely out of people...I think that will take the humanity out of humans. A robot is...doesn’t have empathy, doesn’t have sympathy, doesn’t have trust, doesn’t have friendly approach, it will be just a clear mathematical algorithm thinking, full stop, and this is where everything ends...I have days where you see anxiety, depression, anxiety, depression and so forth, bereavement and so forth—how can somebody like that be able to talk with an AI?

P17; GP; woman

I’ve gone from thinking it’s going to take our jobs and now I’m like actually there’s so much subtlety and nuance and complexity to something like a GP anyway, that I really don’t see us being not needed in the future with AI.

P14; GP; man

Half (10/20, 50%) of the participants expressed that there is a skill and craft that experienced HCPs develop in medical training that involves picking up on nuances and cues through *human* intuition. Although the limitations of guidelines were a major theme in managing MLTC, skillfully navigating circumstances outside usual guidelines was a point of professional pride. Clinicians felt strongly that AI could not replicate this nor take account of complex social factors or mental illness, which were especially relevant for managing MLTC:

A lot of what we do is like a big grey area...I think an AI process maybe couldn’t make those strange decisions [outside usual guidelines] with a patient...when you have a depression review and as soon as you say “hello, how are you”...you can tell straight away, even on the phone you’re like “you sound brighter, you sound better.” And I don’t know if a computer could do that. Or even like picking up non-verbal cues and things where you know...there’s more that they want to tell you so I don’t know if [AI] could deal with the complexities of mental health plus any other condition.

P4; GP; woman

Half (10/20, 50%) of the participants, especially the physicians, were concerned that AI may perpetuate issues they perceived with automated algorithms, which they felt were overly defensive and led to overtreatment. This was based on their experience with patient-facing triage services such as the nationwide NHS 111 service that advises patients on how to manage and receive help for their symptoms:

[The computerized triage service is] completely useless. For really minor stuff...someone’s got like a bump on their skin or something and it will say things like “The rash did not go away when pushed on. It must be meningitis”...and instructed them to go to hospital...The patient’s just like “I don’t really know what that was about, but it was a bit silly.”

P14; GP; man

##### Exciting and Futuristic

In total, 85% (17/20) of the HCPs expressed positive responses, including a belief that AI is powerful and capable of intelligent tasks. These participants felt that AI was the natural next step to using computerized medical records and an exciting, futuristic process that would be especially useful in managing patients with MLTC:

I think in a way the more complex decisions become; the more important AI becomes.

P5; GP; man

It could be really potentially quite helpful and quite useful as a tool in healthcare delivery because...when you’ve got people with multiple conditions, you’re assessing so many different things and trying putting it all together, whereas I’m sure there could be some very fancy computer technology...that would take all that information and put it into some kind of algorithm and say well actually this is what you should be doing, you know, and it doesn’t take away at all from the healthcare professional’s role, it just actually makes our job a lot easier to then enable those decisions with the patient and get the best for them. So, I think it’s definitely the way forward.

P7; pharmacist; woman

##### Dangerous or Inaccurate

Most participants (14/20, 70%) were concerned that AI software could give inaccurate information or not ask the right questions, leading to dangerous or inaccurate conclusions:

I asked [ChatGPT] some questions about managing eye injuries and sent [a consultant ophthalmologist friend] the answers to see what he thought, and he thought some of the answers were a little bit dangerous and not reflective of...best medical practice in ophthalmology.

P6; GP; man

A total of 30% (6/20) of the HCPs (typically those with more experience in research with electronic health records [EHRs]) expressed concern about the quality of coding in primary care EHRs, which may impact the quality of AI-driven analyses:

I think in primary care it’s much more difficult because although we’re very rich in data...you’re reliant upon the coding of individual people and as human beings...what we put into the computer system is what any AI algorithm is going to be using to interpret.

P3; GP; man

One participant expressed concerns specifically about the risk of exacerbating inequalities through bias in the data used to train AI tools. Others did not feel that this was likely to be an issue and even felt that AI might help counteract human biases:

I’ve read a lot about bias as well in AI and again, that kind of ties in very neatly with the health inequalities. So if for example say people who are very affluent come in early for cancer screening and therefore have more prostate cancers diagnosed, then the AI algorithm that we’re not quite sure how it’s determined who’s at risk, might feel that affluent, well-heeled white males are the people who are most likely to have prostate cancer, whereas actually we know it’s not that cohort who are most likely but they’re just the ones who are getting it diagnosed who are coming in.

P14; GP; man

[AI] should hopefully ensure best practice, hopefully, is delivered by everyone everywhere, you know, different biases, you know, different backgrounds, all these different things that affect the healthcare that people receive, so maybe that could be a benefit [of AI] which would be amazing.

P7; pharmacist; woman

##### Inevitable

In total, 55% (11/20) of the participants reflected on the rapid development of AI in everyday life, including social media, search engines, online shopping, generative AI applications (eg, ChatGPT), and driverless cars. They expected that the development of AI in the health care domain is inevitable and may as well be embraced:

I think it’s inevitable really...It’s going to happen, isn’t it?

P18; nurse practitioner; woman

##### Frightening (Existential Anxiety)

A total of 65% (13/20) of the HCPs discussed AI as strange or unnerving, with the potential to cause significant societal changes. Typically, HCPs described concerns they anticipated that other people might have but caveated that they felt that this was more science fiction or conspiracy theory:

There are some really scary fundamental, philosophical questions to be asked for...the future, if you get a...not omnipotent AI but one that can do everything...there’s no reason why that couldn’t happen theoretically, and then that starts to become very, very scary. But I think that’s probably more science fiction.

P14; GP; man

##### Misuse of Data

In total, 35% (7/20) of the participants expressed concern about data security and confidentiality in using an AI decision-making tool:

Was it North Korea who used some software [WannaCry] where they held the NHS ransom for about two days in the 2010...so it’s plausible it could happen again and it might be in the living memory of some patients, so needs to be work done probably to convince them that would handle their data responsibly.

P6; GP; man

#### Behavioral: Minimum Requirements of AI-Enabled Systems in Managing MLTC

##### Management of Public Opinion

All participants (20/20, 100%) felt that public perception of how AI uses health care data would affect the uptake and acceptance of an AI decision-making tool. Many participants (12/20, 60%) commented that older patients often struggled more and objected to the increasing use of technology, particularly in cases in which they did not have wider family support, and that, if patients were expected to interact with the tool (rather than the clinician), then this could perpetuate digital exclusion:

I think there would be some patients that would be very against it because “I don’t trust those things, computers.” So, there would be a lack of understanding and therefore “I’m not signing up to that.”...I think then there’d be another pool of patients who would actually be very much [for it]...our younger cohort, our patients who are into their IT.

P10; nurse; woman

##### Clinician Autonomy

There was strong agreement (16/20, 80%) that there must be an experienced human clinician and informed patient who have oversight of any decision advised by an AI tool. This was felt to be particularly important in MLTC care given the complexity of factors (including sociocultural) contributing to decisions and skepticism that AI would be able to take everything into account:

The clinician has to have the final decision and not just a random algorithm thinking its own algorithmic thinking and then we are feeding—and we become the servants of an AI—I never want to live the days in my careers when I will become a servant of an AI. I want the AI to be used strictly as a tool to enable the existing clinician to do a better job.

P17; GP; woman

I think it’s something you’d definitely discuss with Janet, if she’s happy to proceed...I think it’d be a case of communicating with the patient rather than just taking the advice solely from the AI tool.

P15; GP trainee; woman

Many participants (11/20, 55%) would feel comfortable trusting the output of the AI tool if it suggested a familiar course of action that was in line with usual practice, but others would be wary, especially if the suggestion was unfamiliar or outside their area of expertise:

I’d probably have a high level of trust...I assume it’s gone through the right clinical governance. I don’t double check it because I think you then sort of do away with the value of it.

P5; GP; man

I would prefer to double check myself...[an AI tool] would lead to lazy clinical practice, because you’re not updating or refreshing your own clinical knowledge and practice. And to a certain extent you don’t know how up to date the AI is. So, I’ll be honest, I wouldn’t trust it.

P15; GP registrar; woman

##### Time Efficient and User-Friendly

Participants were clear (16/20, 80%) that AI tools must have a simple user interface and work seamlessly with existing EHRs; otherwise, they would not be used:

As long as it’s not constant clicks...the speed with which you can still make your decision and choose what you’re going to do and...[prescribe] it—that needs to still be very quick or else it won’t be used.

P3; GP; man

##### Clear Rationale

In total, 55% (11/20) of the participants expressed that the AI tool may also take some of the decision-making burden from clinicians and reinforce the decision in cases in which they were not sure. A total of 45% (9/20) of the clinicians stated that they would have more confidence if the rationale behind an AI-based recommendation was explained, especially if the suggestion was unfamiliar. They felt that this could also help in communicating the decision with the patient. For example, in choosing an antidepressant with Janet, they would expect to see the benefits and risk of side effects presented to allow them to help her make an informed choice:

I would be wanting to have a very clear explanation that made me comfortable that that decision was a sensible one and that the balance of risk based on these statistics or whatever was that actually the risk of [Janet] bleeding being on aspirin and sertraline was greater than the increased cardiovascular risk of weight gain or putting up her blood pressure...so it would have to justify it I think or there will be a way of justifying the decision...particularly around MLTC because by definition you’re having to deal with trade-offs—so it’s always helpful when you can have a much more clearer conversation with the patient about what these trade-offs are because ultimately that’s what shared decision making is.

P3; GP; man

##### Clarity of Presentation

Many participants (11/20, 55%) commented that the AI tool outputs should also be easy for patients to understand to allow for comparison of the options and a shared decision. Diagrams and graphic icons (eg, smiley faces that showed the risks associated with each antidepressant option for Janet) were perceived as useful for shared decision-making. Others countered that too much information might be overwhelming for the patient:

Using different fonts...rather than a list of text you could have like a diagram...different colours and things like that, just to make it a bit more interactive...

P7; pharmacist; woman

And if it was able to show you a comparison of mirtazapine, sertraline, perhaps another option as well, and then predicting what might happen to [Janet’s] diabetes, her blood pressure, her depression, comparing those choices...I think knowledge is power, so that would be something that you could use to discuss with the patient. But I’m not sure if that would tend to be a bit overwhelming because you’ve got too much information.

P15; GP trainee; woman

##### Managing Digital Inequalities

Many participants (11/20, 55%) reported that they would use an AI tool with all patients regardless of patients’ demographic characteristics, and 10% (2/20) of the participants described that a potential advantage of an AI tool would be that it may be adaptive and inclusive for patients with additional needs:

...humans are very variable...so I think it would need to be responsive to the different needs of individuals and whether that’s visual impairment, whether that’s learning difficulties or [autism, difficulty with numeracy, patients with multiple physical and mental health conditions]...so I suppose that’s the advantage of AI, is it begins to understand how best to interact with somebody.

P5; GP; man

##### Regulation and Testing

A total of 55% (11/20) of the participants expressed an expectation for AI tools to have a strong basis in research, with transparency in how AI tools operate and rigorous information governance procedures for their introduction into clinical practice, as with any other medical device or risk prediction tool. P16 was involved in assessing and regulating digital tools for health care:

I think it would help to look into studies of how much that helped into reducing the risk of errors, harm to the patient, making right clinical decisions.

P9; pharmacist; woman

[There] is a set of minimum requirements...like legislation good practice and there’s a checklist of things that [NHS England] want [digital companies] to have...it’s really useful—it’s become mandatory for people to do these things.

P16; GP; man

##### Gradual Introduction

Attitudes toward change varied between participants, but half (10/20, 50%) anticipated that a gradual and flexible process would best facilitate the introduction of AI tools into routine practice:

It would probably take a process of time to suddenly change your way of working, change your way of diagnosis and the more you worked with it, the more I guess you would see well actually I like this bit of it, but I actually don’t like that. And that’s just like anything isn’t it?

P10; nurse; woman

#### Environmental Influences

##### Influences Within Society

Most participants (13/20, 65%) recognized that AI is part of everyday life, through search engines, voice-controlled AI assistants (eg, Alexa and Siri), online banking, and online shopping (eg, Amazon). AI was also recognized as influencing social media algorithms. The physicians in particular had tested generative AI tools (ie, ChatGPT) to explore their use in clinical decision-making and searching for clinical information but did not use them routinely. Participants obtained their information about AI from several sources, including science fiction, mainstream news, podcasts, and social media (11/20, 55%), as well as friends and family who worked in or studied IT (6/20, 30%).

##### Influences Within the Health Care Setting

Most HCPs (12/20, 60%) described pressures on the health system as a key driver for using AI in clinical practice. Participants felt under strain to provide good care with limited time and resources, particularly for managing MLTC. AI decision support tools that ameliorated this were welcome:

So, I think it’s definitely the way forward. We’ve got an ageing population, we’ve got more and more conditions, it is complex and difficult, so any kind of help and support with that I think is a great idea...I think there are certain things where you can just apply a computer programme...really quick and simple, great.

P7; pharmacist; woman

Participants who had observed changes in health care delivery over decades were confident that they could continue to adapt. In total, 30% (6/20) of the HCPs referenced changes to health care delivery during the COVID-19 pandemic, with more consultations being delivered via phone or video call:

...we had Covid, that changed many things...It will be likely difficult to accept at the beginning for maybe most people but over the time they maybe get used to it. If it’s helping them.

P20; GP; woman

A total of 15% (3/20) of the participants expressed that they would be most comfortable using AI if it was common practice for other HCPs too:

...as a GP you do worry about making mistakes and getting complaints, so I wouldn’t...be comfortable to use it until everyone was doing it.

P19; GP registrar; woman

Working in a practice with a track record of adopting new technologies made 10% (2/20) of the participants more confident that AI would be successfully adopted in their workplace:

I think our practice would be happy to try new things, but I’m sure some places will be like no, no, we’re not doing that.

P4; GP; woman

#### Individual Characteristics

##### Affinity With Technology

Some participants (5/20, 25%) had a particular interest in AI outside of their clinical role, which influenced their perspectives on AI in health care:

I quite like thinking about the future and enjoyed science fiction and that kind of stuff growing up.

P14; GP; man

##### Independence of Prescribing Decisions

Seniority of decision-making impacted participants’ receptiveness to the introduction of AI decision-making tools. In total, 38% (5/13) of the experienced prescribers (ie, geriatricians and qualified GPs) felt that they would use an AI tool differently from allied health professionals and registrars:

One size will not fit everybody...if you give it to a...GP and if you give the same one to a...ACP [advanced clinical practitioner (ie, nurses, pharmacists, and physician associates)]...that AI will tell you “no do this” and you know...that can be like bypassed those things sometimes. But for a person who is much more junior they need to go through a step, so yeah, that has to be taken into consideration—who is using it and who has to use it because that will make a huge impact on the acceptance of its use.

P12; geriatrician; man

One GP (P17) was concerned that physician associates using AI might not provide all the necessary inputs to an AI tool or be able to judge the validity of the output:

I would not be 100% happy...If my [physician associate] colleague will diagnose for example a cough being a respiratory condition but if they haven’t asked x, y and z question and they have not eliminated the other list of two or three diagnostics, then I will not be happy for an AI to interpret just the data because it means that that patient history taking was not sufficient enough for an AI to create that algorithm.

P17; GP; woman

In total, 57% (4/7) of the prescribers who practiced less independently, such as GP registrars and nurses or pharmacists, anticipated using the tool as an alternative to seeking advice from a senior clinician:

So many practice nurses are working on their own. In hospital you’ve got a team, haven’t you? And now more than ever in General Practice, you haven’t got that time to be knocking on the door, waiting for [a GP to get advice from]...so it would speed up and I suppose increase our confidence as well.

P10; nurse; woman

## Discussion

### Principal Findings

We identified 3 main themes that summarize the challenges that HCPs perceive in managing MLTC: first, the difficulty of balancing multiple factors, including managing mental and physical conditions and accounting for patients’ social circumstances; second, managing the burden and risks of polypharmacy; and third, practicing outside of single-condition guidelines. We then used the model by Buck et al [23] to delineate and summarize the cognitive, affective, and behavioral components of HCPs’ attitudes toward using AI tools to manage MLTC. HCPs thought that AI tools would improve the safety and efficiency of decision-making with patients with MLTC by facilitating the process of weighing the multiple factors to be addressed. The potential for AI tools to change the clinician-patient relationship was a universal concern for all participants, and they discussed whether these tools would reduce the interaction between clinician and patient and how they would cope with nuance or gray areas inherent in supporting people with complex medical and social circumstances. We identified several minimum requirements for AI tools, as perceived by HCPs, that have key implications for their successful implementation in clinical practice, including public perception of these tools, the importance of time efficiency, and the fact that clinicians and patients with MLTC must retain autonomy over important medical decisions.

### Comparison With Other Research

In total, 2 systematic reviews including studies of GPs’ perspectives on the management of patients with MLTC reported similar themes to those we identified (although these studies did not have a focus on use of AI tools in this context) [29,30]. These studies, covering 15 mostly high-income countries, report, as we do, that GPs found the limited evidence base and single-condition guidelines to be inadequate in managing MLTC [29,30]. The importance of patient-centered care was a key theme in both reviews, as well as the fact that the challenge of MLTC management is often compounded by patient characteristics, including memory problems, poverty, and poor health literacy and social support [29]. It is notable that these themes were consistent across a range of countries, although these settings typically had a similar model of general practice to that of the United Kingdom [29].

To our knowledge, ours is the first study to examine the attitudes of HCPs toward using AI decision-making tools in the context of managing MLTC. HCPs in our study typically did not feel that they fully understood AI but were optimistic about its potential to improve the safety and efficiency of decision-making with patients with MLTC. Participants in our study felt that the use of AI-based tools would be similar to existing non-AI decision-making tools and perceived that existing regulatory processes would ensure that AI tools were safe for practice. Terry et al [31] reported a similar response from Canadian health care stakeholders, who commented that the “mechanics of AI don’t matter [it’s] just another tool in the toolbox.”

The model by Buck et al [23], used to interpret the determinants of German GPs’ attitudes toward AI in general practice, provided a useful lens to understand the determinants of our participants’ attitudes toward AI. Their findings overlapped with ours—we also found that HCPs’ expectations and concerns fed directly into the minimum requirements for an effective AI decision support tool. Both our study and that by Buck et al [23] placed large emphasis on the perceived risk to the clinician-patient relationship. However, while the study by Buck et al [23] described GPs’ perception of the “lack of human competencies” in AI systems as an *expectation*, we interpreted the emphasis on the skill and craft of clinicians and the fear of human interaction being replaced as a deeply felt affective *concern* regarding something that potentially threatened participants’ professional pride. GPs especially placed high value on the art of picking up on nuanced cues from patients, deducing when the guidelines are not appropriate for an individual, and delivering a therapeutic human interaction—skills that clinicians must develop through years of clinical training. These factors were identified as integral to the challenge of managing MLTC; thus, while AI decision-making tools were considered to have exciting potential, HCPs felt that they must be introduced judiciously.

Our findings broadly aligned with those of the exploratory overview by Scott et al [32] of 27 studies on stakeholder views on AI in clinical practice. They reported, as we do, that clinicians were in favor of AI applications that summarized data or interpreted results. A survey of UK GPs by Blease et al [33] found receptiveness to using AI to reduce administrative task burden in primary care. Both papers reported that empathy and communication were essential components to primary care, and physicians were skeptical that technology would be able to replicate effective information gathering or caring interactions with patients. A further survey of Korean hospital physicians showed that they also doubted that AI could replicate the “human touch” and had concerns about who carries the legal liability for decisions [34]. Scott et al [32] also report that clinicians were worried about privacy breaches and who bears liability for AI-supported decisions. However, these concerns were raised by only a minority of our participants, possibly because the use of the AI tool was contextualized through our case study as a familiar process, not dissimilar from using existing computerized decision support tools.

Those developing AI decision-making tools for use in primary care should be aware of the minimum requirements reported by our participants. The influence of patient and public perspectives on AI in health care on the participants’ willingness to use it was expressed by all participants in our study, which may reflect that participants were specifically asked about this in our interviews. Similarly to the studies by Blease et al [33] and Buck et al [23], we conclude that general practice is under high demand and that time efficiency and integration with existing technology would be essential to the uptake of AI in clinical practice. The model by Buck et al [23] had diagnostic accuracy as a minimum requirement. Participants in our study were also concerned about the danger of AI providing wrong answers, but potentially because of our focus on MLTC, which was described by participants as a *gray* area outside usual guidelines, we felt that the emphasis in terms of clinician confidence in using AI was less on the fact that it gave the “correct” answer and more on the fact that clinicians could appreciate the rationale for the decision advised by the AI tool. For AI tools to be used in MLTC care, the rationale behind a recommendation should be apparent, which may encourage further learning for the HCP and facilitate communication with the patient. Buck et al [23] found, as we did, a strong consensus that a hybrid clinician-AI service may combine the strengths of both clinicians and AI tools. Although AI could support the decision-making process, HCPs strongly believed that they (and their patients) should make the final decisions.

In presenting these findings to clinical academics at conferences, audience comments included concerns about the risk of AI perpetuating biases inherent in the underlying data and causing inequality in care provision. Research has shown that, in cases in which AI tools are trained on data that lack representation from people with marginalized characteristics, such as their socioeconomic status, age, ethnicity, gender, or sexual orientation, this can lead to disparities in health outcomes and accessibility [11]. In a Canadian qualitative study in which discussions were facilitated between primary care HCPs and patients, concerns about bias were raised in every session by both parties [35]. Surprisingly, only one HCP participant in our study expressed concern about this (although 14/20, 70% of the participants were concerned about inaccuracy of the AI tool in general), and some participants felt that AI tools might instead help health care services adapt to patients with diverse needs, which is often the case in those with MLTC. However, 60% (12/20) of the participants were concerned about digital exclusion, particularly for older patients, if patients were expected to interact with an AI tool directly.

Buck et al [23] found that age could influence attitudes toward AI. Some participants in our study did have concerns regarding whether older patients would be receptive to AI tools and the fact that they would be more at risk of being digitally excluded from health care. However, while the age of the participants did not appear to correlate with their attitude toward AI tools, we noted some indications that clinicians may use AI tools differently depending on their level of experience in prescribing and whether they are accustomed to autonomy in their clinical decision-making. Experienced GPs and geriatricians were more inclined to override an AI tool in favor of their own knowledge, whereas GP registrars and newly qualified pharmacists considered the outputs of the AI tool as educational. This correlates with the findings of a study on AI-facilitated decision aids for radiology, which found that less experienced clinicians were more likely to trust AI advice than clinical experts [36].

### Strengths and Limitations

The use of an existing theoretical framework with both inductive and deductive analyses allowed for a deeper and more comprehensive understanding of attitudes toward AI in the context of MLTC. Most previous studies exploring perspectives on AI have approached it as a hypothetical idea without a real-world example (likely because few exist), which may limit the depth or specificity of their discussions [12]. Using the case study of Janet helped ground our participants’ responses in the context of their usual practice in managing MLTC and elicit more detailed reflections with meaningful insights into how the use of the OPTIMAL tool may change their consultations. The participants were interviewed by a “fellow” GP, which allowed for an in-depth discussion of the technical aspects of current and future use of technology in decision-making. Input from our public advisory group throughout the study also helped ensure that the interviews and analyses followed the intended study objectives.

We interviewed every HCP who was willing to participate, so we were not able to be selective about the demographics of the participants. However, our participants’ ethnicity and gender profile is broadly reflective of that of GPs in the West Midlands (GPs in England are just over 50% female, as was the case with our sample; in our sample, 7/20, 35% were White individuals, and 8/20, 40% were Asian individuals, whereas 56% of GPs in England are White individuals and 25% are Asian individuals). The participants were slightly younger than average GPs in England; no participants were aged >55 years, and 50% (10/20) were aged between 35 and 54 years, whereas 54% of GPs in England are aged 41 to 60 years [37]. A recent survey of the UK general public found that women and those aged >55 years are more likely to have concerns about the use of AI [38]. Although we did not find age to correlate with attitudes toward AI, a limitation of our study is that it did not include the perspectives of the oldest working HCPs, who might be more skeptical than younger HCPs.

This convenience sample of busy HCPs in the West Midlands of the United Kingdom willing to give their free time to be interviewed might have attracted those with either a vested interest in AI or more extreme opinions opposing it. The focus on this region limits comparison across regions or with international participants. This may affect the wider generalizability of these findings, which may be most applicable in the UK NHS context. However, as Smith [39] highlights, qualitative research typically seeks naturalistic rather than statistical generalizability. We provide rich, contextualized insights that are likely to resonate with and inform other settings. Our study contributes to broader conceptual understandings of how AI might be integrated into complex care settings and offers transferable learning for similar health systems and policy contexts.

A total of 10% (2/20) of the GP participants were known to the interviewer before this study through being in the same training program; it is possible that this influenced how they responded to the questions. The interviewer effect, which describes the influence of interviewer characteristics and behavior on participant responses, is an established phenomenon in qualitative research [40]. There are mixed perspectives on whether previous rapport through preexisting relationships reduces or increases social desirability bias [41]. Social desirability bias, whereby participants might tailor their responses to those that align with socially accepted behaviors or values, is known to influence responses in qualitative studies [42]. In our experience within this research, previous rapport seemed to facilitate more engaged, honest responses with less apparent social desirability bias. To ensure full transparency, participants were aware that the interviewer was part of the OPTIMAL study, which aims to develop an AI decision support tool, so they could have felt compelled to discuss AI more positively than how they actually felt. It is possible that we overestimated optimism about AI among HCPs, and given that we saw less engagement than expected with the issue of the effect of AI on inequalities, it is possible that participants were influenced by (lack of) social desirability in discussing biases. However, perspectives on the use of AI in managing MLTC are unlikely to be considered highly sensitive or stigmatizing. We mitigated this potential effect by reassuring participants that their responses were anonymous and prefacing each interview with the sentiment that “there are no right or wrong answers, we are simply interested in your thoughts,” as well as through the use of a standardized topic guide with questions framed in a neutral manner.

### Implications for Practice, Policy, and Research

Our findings are important to software developers, HCPs, and policy makers in navigating the development and regulation of AI tools for managing MLTC. There is currently limited guidance on how AI tools should be safely integrated into clinical practice, and clarity on the medicolegal implications for clinicians of using these tools is urgently needed [9]. Patients with MLTC present complex care needs, including difficulties prioritizing multiple conflicting needs, managing polypharmacy, and navigating care outside single-condition guidelines. These challenges highlight the need for AI tools that are not only technically robust in dealing with multiple factors but also user centered and responsive to real-world complexity. Patients’ perspectives are especially important to HCPs’ confidence in using AI in the consultation. Further research on the perspectives of patients with MLTC on AI-based decision-making tools is needed.

The minimum requirements for AI tools that effectively support clinicians and patients in managing MLTC are as follows: (1) integration with existing EHR systems to promote time efficiency and reduce workflow burden; (2) gradual introduction to support clinician and patient trust; (3) transparency in the rationale for recommendations that accommodates the complexity of polypharmacy and comorbidity management to support clinicians in adjusting treatment plans beyond single-disease guidelines; (4) preservation of clinician autonomy, allowing for adaptation to individual patient needs; (5) presentation in a patient-friendly format that supports shared decision-making; (6) design for accessibility across diverse patient and practice contexts to avoid exacerbating digital inequalities; and (7) rigorous medicolegal regulation and real-world testing.

### Conclusions

HCPs believed that AI tools will be effective in improving the safety and quality of decision-making in the complex domain of managing MLTC. However, they were concerned that AI may lack the nuance and humanity that are fundamental in supporting people with complex medical and social circumstances. We identified critical minimum requirements for the adoption of AI, including seamless integration into existing systems, transparency in AI decision-making processes, and the retention of clinician and patient autonomy over medical decisions.

## Appendix

Multimedia Appendix 1 Interview topic guide.

Multimedia Appendix 2 Information sheet for participants.

Multimedia Appendix 3 Background information on artificial intelligence for participants.

## Abbreviations

AI: artificial intelligence
EHR: electronic health record
GP: general practitioner
HCP: health care professional
MLTC: multiple long-term conditions
NHS: National Health Service
OPTIMAL: Optimising Therapies, Disease Trajectories, and Artificial Intelligence–Assisted Clinical Management for Patients Living With Complex Multimorbidity
