# Supplementary material for: Perspectives of Health Care Professionals on the Use of AI to Support Clinical Decision-Making in the Management of Multiple Long-Term Conditions: Interview Study
Source: J Med Internet Res. 2025 Jul 4;27:e71980. doi: 10.2196/71980 (PMC12274781; doi:10.2196/71980)
Supplement: Multimedia Appendix 1 [file jmir_v27i1e71980_app1.docx]

**
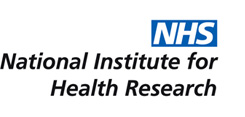

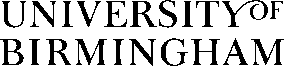
**

**OPTIMIsing therapies, disease trajectories, and AI assisted clinical management for patients Living with complex multimorbidity (OPTIMAL):**

**Interview guide: Staff Interview**

**This is the starting topic guide. The overarching objectives will remain the same, but questions and prompts will be developed as interviews are undertaken to incorporate any important themes emerging from the interviews.**

**Before the interview begins**

- Ensure the participant has read the information leaflet
- Ensure the participant has had the opportunity to ask any questions about the research including issues about confidentiality, the findings of the research and where the research will be disseminated before being asked to agree to each item on the consent form.
- Start audio-recording
- Go through each item on the consent form and record their verbal consent. Explain that you will send/email a copy of the consent form for their records. They should already have a copy of the participant information sheet with details about the study, how to withdraw etc
- Explain that they don’t have to answer all the questions just because they have consented to the interview, and that they can take a break or stop the interview at any time.
- Explain that you are there to understand more about their experiences and that they will have some time at the end of the interview to talk about any other issues that are important to them that may not have been covered by the questions.
- Check that they are happy to continue to be audio-recorded.
- Begin the interview.

**Topics to be covered in the interview**

Managing patients with four or more long-term conditions?

**What are your experiences of managing patients with four or more long-term conditions?** Prompt – what factors are important to consider when compared with patients with single diseases , comorbidties, other meds

**What difficulties do patients with four or more long-term conditions have in managing their conditions and making decisions about treatment options?**

Acceptability of AI in clinical decision making

**Attitudes and understanding about AI in general terms** (briefing guide of discussion points about AI) such as Amazon/ BBC iPlayer/ Spotify giving recommendations based on the existing data they have collected?

**Do you have any experience of using AI in clinical practice?**

Attitudes towards and perspectives about AI health care in managing cMM.

**How do you think using AI directed clinical decision making may compare to usual practice?**

Thinking about the impact on health care professionals ***and*** patients:

**Thinking specifically about managing physical health conditions: Prompt? Diabetes, COPD, heart failure, CKD:**

**What are your thoughts on advantages and disadvantages of AI directed clinical decision making compared with your usual practice?**

**How do you think patients feel about AI directed clinical decision making?**

**What do you think patients may see as the advantages/ disadvantages of AI directed clinical decision making?**

**Are there examples where either using AI or using usual practice might be preferable?**

**What kind of things may influence your choice?**

**What are your thoughts on using AI directed clinical decision making with patients who have mental health conditions, e.g. depression/ anxiety?**

**What are your thoughts about using AI directed clinical decision making to make decisions about prescribing in physical health conditions (e.g., choosing the best medication for diabetes).**

**What are your thoughts about using AI directed clinical decision making to make decisions about prescribing treating mental health conditions (e.g., choosing the best antidepressant).**

**How confident would you feel if prescription decisions were directed by a predictive algorithm compared to your usual practice?**

Very comfortable Mostly comfortable Not sure Mostly uncomfortable Very uncomfortable

**Why?**

Stage two: Vignettes – validity of AI in clinical decision making

We will present to the participant, a range of simulated patients from a range of disease clusters, to compare how aspects of the AI patient care management fit in with their own experiences a clinician:

**What resources would you normally use to help make complex decisions like this?** (NICE guidelines, own experience, other guidelines)

**What medication changes would you recommend and why?** (pt's age, sleep/appetite problems, other health problems, other medications, patient preference)

**The computer algorithm says the patient should take** X with the following explanation**, do you agree?**

**How would you feel about using the computer algorithm to make a decision like this compared with your usual practice?**

Very comfortable Mostly comfortable Not sure Mostly uncomfortable Very uncomfortable

**Why?**

**How would you feel if the algorithm recommended a treatment you were not expecting?**

**Would you over-ride if you disagree?**

**What would you do if a patient didn’t want to follow the recommended treatment (i.e., would you look to over-rule it or stick by it?**

Close of interview

Thank you. That was my last question. Is there anything you would like to add about the things we talked about but have not covered in the interview?
