# Supplementary material for: Perspectives of Health Care Professionals on the Use of AI to Support Clinical Decision-Making in the Management of Multiple Long-Term Conditions: Interview Study
Source: J Med Internet Res. 2025 Jul 4;27:e71980. doi: 10.2196/71980 (PMC12274781; doi:10.2196/71980)
Supplement: Multimedia Appendix 3 [file jmir_v27i1e71980_app3.docx]

**Artificial Intelligence (AI) Background for participants**

Artificial Intelligence or AI is the term used to describe a computer system or algorithm that can conduct tasks that would normally require human intelligence.

There are examples of AI being used in our everyday lives, with applications and software such as Spotify, Amazon and BBC iPlayer. These ‘apps’ and on-line websites function by predicting what kind of music, TV programmes, or general purchases we may like or want. They do this by using a computer programme that can observe what categories and kinds of TV, music etc. we watched or bought in the past and use this data to predict what we are likely to choose in the future.

AI is also used in things like self-driving cars and language translations, and it is also being increasingly developed for use in healthcare. For example, AI has been developed that can screen the retina scans of patients with suspected diabetic eye disease. This is the leading cause of blindness in adults, and in many parts of the world, there are not enough doctors or health care professionals to undertake the work involved in diagnosing the condition. The AI trained system can diagnose the condition with the same accuracy as a trained healthcare professional.

In healthcare AI is also being developed to help us to predict how patients’ health may progress in the future. It can also help to decide what are the best treatments and medications for the increasing number of people who are living with several long-term health conditions

Using the large amounts of anonymised patients' data available from GP and hospital electronic health records, AI can help us predict the life trajectories of people with multiple long-term conditions. For example, we know that people with diabetes may already have or are more likely to develop high blood pressure, heart disease and eye disease. These patients are usually prescribed medications to help manage all these conditions, but it can be difficult to ensure the best medications are prescribed for each individual patient based on the complexities of their own specific medical history, other characteristics and the variability in decisions made by different health professionals providing care.

Given the variability of factors that can influence health, having an efficient, accurate and easy to use AI programme that can take into account this variability and help ‘predict’ the best combination of medications, or what conditions may develop in the future may benefit patients and health care professionals.
